# Febrile Seizures in Children: A Review

**DOI:** 10.7759/cureus.31509

**Published:** 2022-11-14

**Authors:** Aakriti Tiwari, Revat J Meshram, Rakshit Kumar Singh

**Affiliations:** 1 Department of Paediatrics, Jawaharlal Nehru Medical College, Datta Meghe Institute of Medical Sciences, Wardha, IND

**Keywords:** epileptogenesis, febrile seizures, febrile-associated seizures or epilepsy (fase), temporal lobe epilepsy, dravet syndrome

## Abstract

Fever-induced seizures are referred to as febrile seizures (FSs). The most prevalent kind of epilepsy and neurological illness in infants and young children is FS. With a high occurrence seen between the ages of 12 and 18 months, they frequently affect children aged six months to five years. FS is a benign condition that seldom results in brain damage. Nevertheless, they cause stress and emotional anguish for the parents, who may believe that the death of their child is going to occur during the seizure. Lately, a more broad-based phrase has been used, fever-associated seizures or epilepsy that includes simple, complicated, and extended FSs. These are the three different kinds of FSs. Febrile status epilepticus is a subgroup of complex FS. The other kinds of FSs are FS plus, Dravet syndrome, hereditary epilepsy with FS plus, and febrile infection-related epilepsy syndrome. The most frequent, brief, and generalized simple FSs have a greater likelihood of causing temporal lobe epilepsy than complex FSs. These seizures are linked to the release of inflammatory mediators like interleukin (IL)-1, IL-6, and tumor necrosis factor, which are well-known fever inducers. This article details the factors that contribute to the occurrence of FSs, epidemiology, pathophysiology, evaluation, and management of the child.

## Introduction and background

The most frequent seizure type, known as febrile seizures (FSs), affects 2-5% of children under five years of age, with a peak incidence in the 24 months of life [1]. FSs affect one in 20-50 children, making them the most common type of aberrant brain activity throughout development [2]. The International League Against Epilepsy (ILAE) defines FSs as those of children between the ages of six months and five years who were formerly afebrile and experienced febrile illness-related seizures without a recognized cause (such as an infection of the neurodevelopment system, a metabolic disease, trauma, or intoxication) [3]. As febrile sickness is thought to be a trigger of an underlying propensity to epilepsy, children experiencing FSs are not included in the category of children who have had no prior FSs [4,5]. The National Institute of Health (NIH) advocated an age limit earlier, but the ILAE definition's lower age limit is younger. According to the NIH consensus conference, FS is a condition that typically affects children aged three months to five years and is accompanied by a fever but lacks any indication of intracranial infection or a known etiology [1,6]. Seizures associated with febrile illness are categorized based on the length and frequency of convulsions [7,8]. The various differences between simple, complex, and extended FSs are mentioned in Table 1 [9,10]. Simple FSs, which typically last 15 minutes, are caused by a particular illness, like a respiratory or digestive infection [9]. More than one complex FS occurs throughout each episode of fever, and their life period is between 15 and 30 minutes [10]. The term febrile status epilepticus (FSE) is commonly used to describe seizures that last more than 30 minutes. FSE, despite making up a very tiny part of FSs, accounts for 25-52% of all instances of status epilepticus in children [11,12]. Up to 41% of children who have FSE go on to experience another FS in the future, increasing their risk of other negative outcomes [7]. A larger risk of FSE exists among children with underlying neurological disorders, who comprise over a quarter of all FSE cases [11]. The condition known as febrile infection-related epilepsy syndrome, which is comparable to FSs, affects young children between the ages of three and 15 years. However, the seizures are similar to complex seizures in children in that they last 15-30 minutes or, in exceptional cases, enter status epilepticus and stay longer than 30 minutes [13].

**Table 1:** Simple FSs differ from complex and extended FSs. FS: febrile seizure

	Simple FS	Complex FS	Extended FS
Onset	During bouts of extreme febrility	Throughout mild-to-moderate febrile bouts	Intermittent or ongoing FSs
Duration	Few minutes	Prolonged (10 min)	Nil
Type	Generalized tonic-clonic seizures	Hemilateral or focal	Nil
Other FS	Recurring	Most recurring	Nil
Postictal events	None	Some lingering Todd’s paralysis	Nil
Previous neurological signs	Absence	Potential presence	Nil
Clinical outcome	Decent	Afebrile seizures 10–15% cases	Nil

Methodology

The methodology of this research is an exhaustive review of the literature searched from PubMed, Elsevier, ResearchGate, Medline, Embase, The Cochrane Library, and other important databases.

Epidemiology

Between the ages of five months and five years, FSs are most common, peaking at 18 months. About 20-30% of FSs are complex, with the majority being simple [14,15]. In comparison with the general population, families of children with FS experience an increased rate of epilepsy. According to the results of one study, 9.2% of FS patients had first-degree relatives who had epilepsy [16]. The occurrence of FS in kids in West Europe and the United States varies from 2% to 5%, and the maximum age when it appears is 18 months [17,18]. Kids from various ethnic backgrounds may present with FS. However, some populations have a higher prevalence than others, consisting of Japanese (6%) and Indians (5-10%) [18]. Fifty percent of all children with FS are between the ages of 12 and 30 months, while just 6-15% of children encounter their first episode after turning four [19]. 

## Review

Etiology and pathophysiology

It has been established that specific viruses can cause FSs. Influenza both A and B, respiratory syncytial virus, adenovirus, human metapneumovirus, parainfluenza viruses 1, 2, 3, 4a, and 4b, rhinovirus, rotavirus, human herpes virus 6, enterovirus, and human metapneumovirus are the viruses most commonly linked to FSs in young children. Upper respiratory tract infections (URTIs) and the common viruses that cause URTIs, such as influenza viruses and respiratory syncytial virus, have been related to FSs in numerous studies. This association is supported by the fall/winter seasonality of these events [20,21]. Peaks in the frequencies of FSs are correlated with an increase in summertime diagnoses of gastroenteritis in children, particularly enteroviruses [22]. Various vaccines, such as the rotavirus vaccine, measles mumps rubella vaccine, diphtheria pertussis tetanus vaccine, and influenza virus vaccine, are correlated with the generation of FSs. Although the specific pathogenesis of FSs is uncertain, it is usually accepted that those events are caused by a confluence of environmental exposures (the fever and its cause) and genetic predisposition.

Fever and Seizure Induction

**Figure 1:**
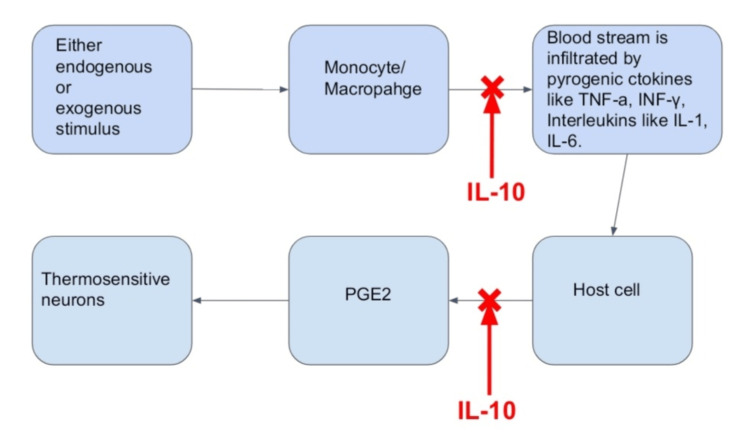
A condensed version of the pro-cytokines that promote an anti-inflammatory as well as the febrile response. TNF-a: tumor necrosis factor alpha; INF-γ: interferon gamma; IL: interleukin; PGE2: prostaglandin E2. Image credit: Authors.

According to a theory, increasing brain temperature will promote the firing of neurons and increase the likelihood of synchronized activity of neurons, which causes seizures [23]. Stressors throughout pregnancy and the first few months after birth may influence limbic epileptogenesis by altering the neuroplasticity of the central nervous system [24]. By drawing astrocytes and microglia to the location of the lesion, early life trauma is thought to change the excitability of circuits (for example, infection to the mother, prenatal maternal or environmental stress, perinatal hypoxic-ischemic injury or any infection after birth of the baby, seizure, or trauma leading to brain injury). After this reduction in seizure threshold in the growing brain, a second hit (like, say, a fever) can be sufficient to trigger a seizure [25]. The immune system's pyrogenic pathway, which produces the cytokines interleukin (IL)-1, tumor necrosis factor alpha (TNF-α), IL-6, and interferon, can also be activated by fever. The anti-inflammatory cytokine, IL-10, is also produced in response to IL-1, IL-6, and TNF-α [26]. IL-1β and IL-10 are increased in FSs [26,27]. Prostaglandin inhibiting antipyretics do not shorten the length or frequency of FSs. This demonstrates that prostaglandins (generated as a result of cytokine release in the fever response, as seen in Figure 1) [4] do not trigger seizures in febrile illnesses [28,29].

Genetic Predisposition

Studies on twins and families suggest that the etiology of FSs has a significant genetic component febrile convulsions 1 (FEB1). A single large family's parametric linkage analysis has revealed the locations of FEB1 on chromosome 8q13-21, febrile convulsions 2 (FEB2) on 19p133, and febrile convulsions 3 (FEB3) on 2q23-24. An assortment of 47 tiny families was the subject of a nonparametric assessment, and febrile convulsions 4 (FEB4) was discovered on 5q14-15 [30]. Voltage-gated sodium channel alpha subunit 1 (SCN1A), voltage-gated sodium channel alpha subunit 2 (SCN2A), and gamma-aminobutyric acid (GABA) receptor subunit encoding genes have mutations in FSs. In 25-40% of cases where a child comes with FS, a positive familial history of FS can be found [31,32]. A sibling's likelihood of developing FS depends on how many FSs a child has. In numerous twin registries, monozygotic twins have significantly greater concordance rates for FS than dizygotic twins [33]. Children who have FS and later develop generalized epilepsy without having a particular epilepsy condition may be explained by the phenotype of FS plus. These individuals or members of their families have a background of FS, which is generally complex and emerges after the age of five. Epilepsy can manifest later in childhood or adolescence in a variety of seizure types. These families have been linked to mutations in SCN1A, SCN1B, and gamma-aminobutyric acid type A receptor subunit gamma2, among other genes. The clinical epilepsy phenotypes that make up the putative genetic syndrome known as generalized epilepsy with FS plus (GEFS+) range from mild to severe, with myoclonic astatic epilepsy being the most severe [34,35]. There are two mutations that relate to the genetic susceptibility of FSs.

Voltage-Gated Sodium Ion Channels

In neurons, voltage-gated sodium channels play a major role in the spread of action potentials. It is supported by the invention of multiple subtypes of sodium channel that there are variances in the genes responsible for producing the sodium channel protein [36]. SCN1B gene mutations have been discovered in all people with seizures, including FSs, in studies of families with GEFS+ [37]. Additionally, a recent study linked two families with GEFS+ syndromes to mutations in the SCN1A gene [38]. Other SCN protein mutations have been discovered and are demonstrated to exist in families with similar phenotypes [39]. However, GEFS+ is a rare cause of FSs, making it challenging to pinpoint the precise impact these mutations play.

Hyperpolarization-Activated Cyclic Nucleotide-Gated Channels

The development of seizures depends on hyperpolarization-activated cyclic nucleotide (HCN)-gated channels, which promote the excitability of neurons [40]. In individuals with seizures and epilepsy, mutations to the hyperpolarization genes activate cyclic nucleotide-gated channels, supporting their function in neuronal excitability. Various seizure disorders, such as FSs, have been linked to HCN1 mutations [41]. Voltage-gated sodium channels are principally responsible for the propagation of an action potential in neurons. It is supported by the discovery of multiple sodium channel subtypes that there are variances in the genes responsible for producing the sodium channel protein [42]. Additionally, patients with FSs and GEFS+ persons have specific HCN2 channel mutations but not those with idiopathic generalized epilepsy, suggesting that HCN2 channels play a special role in attacks triggered by fevers [43].

Risk factors

Genetic predisposition is a significant factor of risk for the occurrence of FSs, as was mentioned before in the text. Other important risk factors are briefly addressed below.

Intrauterine Risk Factor

In an inhabitant, prospective questionnaire survey that began in early fetal life and included 3,372 participants, the occurrence of FSs was evaluated at the ages of 12 and 24 months [44]. Compared to children in the highest percentile, children in the lowest percentile of transverse cerebellar diameter in the second trimester had a higher chance of having FSs. Children with general growth characteristics (femur length, abdominal circumference, and estimated fetal weight) in the third trimester were more likely to develop FS. Children with biparietal diameters in the third trimester's lowest percentile also had a higher probability of developing FS. The study concluded that fetal growth retardation increases the risk of FS and that unfavorable environmental and genetic factors during pregnancy may play a role in the causation of FS. Low birth weight and a brief gestation have also been shown to be important risk factors for FSs by the Danish Birth Cohorts (Aarhus Birth Cohort, Aalborg-Odense Cohort, and Danish National Birth Cohort) [45]. 

Metabolic Abnormalities and Vitamin Deficiency

Some statistical association has been reported between iron deficiency anemia and simple FSs. When compared to age-matched febrile children without seizures, patients with FSs in India had lower zinc and iron levels [46]. Independent of the severity of the underlying infection, several studies have suggested a relationship between FSs and systemic respiratory alkalosis. To find out whether these associations can be important in prevention or if they can be predictive, large population studies are needed [47]. 

Neurological Deficits 

Children with underlying neurological impairments, such as cerebral palsy or neurodevelopmental delay, are more likely to experience FSs. Discharge of the neonate at 28 days of age or later, maternal smoking, and stress are three environmental risk factors linked to an increase in the frequency of FSs.

Evaluation

The necessity to use anticonvulsants such as midazolam or diazepam to halt the seizures is decided on the basis of factors such as the type and length of the spasm, the presence and duration of the postictal phase, recent history of fevers or infections, the use of antibiotic treatment within the last few weeks, any additional accompanying symptoms, and immunization as well as vaccination history. The various other factors that should be kept in mind are past history of occurrences of FS or perhaps a known case of epilepsy, the severity of the convulsions, usage of antipyretic medications, family history of FS, epileptic, or neurological abnormalities. The child needs immediate stabilization utilizing the ABCDE (examination of the airways, breath, circulation, disability, and exposure) strategy, in addition to a blood glucose check, if they are still convulsing and should halt the seizure as soon as feasible using antiepileptic medications. After stabilization, it is important to keep an eye on vital signs such as blood sugar, capillary refilling time, temperature, heart rate, breathing rates, and others [48]. Meningitis and encephalitis are two intracranial infections that must be ruled out as soon as possible in young children since their signs and symptoms can be quite mild [49]. Investigations should seek to identify the etiology when a patient presents with fever and seizures because there are many potential differential diagnoses. If there are no other symptoms, there should be no testing done for simple FSs. Following a good workup, a diagnosis of exclusion for complex FSs is frequently made [50]. The therapy of FSs is not typically changed by a full blood count, serum electrolytes, calcium, magnesium, phosphorus, or blood glucose levels. These should only be carried out if additional clinical signs, such as protracted postictal sleepiness or suspicion of bacteremia, indicate the need for these tests.

The American Academy of Pediatrics has guidelines for examining the first simple FS, which suggests that when a child appears within 12 hours of a simple FS, doctors should try to determine the cause of the fever. The chance of bacterial meningitis manifesting as a first simple FS is thought to be extremely low, and the recommendations mentioned earlier may not be carefully adhered to, according to recent United States studies. On the other hand, they may not as easily follow these evaluation rules in nations where diseases like malaria are more prevalent. It is frequently challenging to distinguish between the presence and absence of intracranial infection in children with malaria who have a fever and complex FS [51]. 

Lumbar Puncture 

A lumbar puncture should be performed when the clinical history indicates meningitis, barring contraindications like signs of elevated intracranial pressure with altered consciousness, focal neurological signs, cardiorespiratory compromise, a bleeding disorder, or infection in the region the needle will traverse. If there is a medical condition that prohibits lumbar puncture, antibiotics should be started [52]. Meningitis is uncommon in children who appear with convulsions and fever in the developed world (0.23%). Nevertheless, 24% of children with meningitis get seizures. A lumbar puncture is not essential in most children since meningitis may be ruled out based on clinical evidence. According to population-based prospective studies, up to 18% of children with febrile status epilepticus develop bacterial meningitis. Thus, early parenteral antibiotics in febrile status epilepticus have been advised, followed, if safe, by a lumbar puncture. In children older than two years, meningitis is extremely rare in the absence of complex FSs, meningeal irritation, or petechiae. Other symptoms, such as being sick for a few days, vomiting, tiredness, decreased feeding, or complex FSs, are typically present in children under two years who have meningitis without meningism. However, in young children who may not exhibit meningism or any child who frequently seeks medical attention, there needs to be increased awareness of meningitis [53]. 

Electroencephalography

To rule out the presence of neurologic disorders, children with a history of complex or recurrent FS or who come with neurological abnormalities may be subjected to electroencephalography (EEG), computed tomography, magnetic resonance imaging (MRI), or a combination of these. An EEG is not advised after an FS in a healthy child with a clear source of infection. If an EEG is taken, it should be taken at least 48 hours after the FS to prevent conflating postictal electrical activity with aberrant activity. It is also discovered that EEG slowing was not a reliable indicator of epilepsy. Therefore, it is unlikely that early EEG abnormalities following a first complex FS will reveal patients at risk for epilepsy. The age of the patient, the time of the EEG, and hereditary diseases are likely just a few of the variables that can affect the development of EEG abnormalities [54]. Children who exhibit recurrent bouts of simple FS and have a clear source of infection do not require a repeat of this test, but it is crucial to identify the infection's cause and manage it correctly [55].

Neuroimaging

In children with simple FSs, neuroimaging is not required. Children who present with complex FSs and are neurologically otherwise healthy are unlikely to have serious intracranial pathological conditions that require immediate neurosurgical or medical intervention, such as a space occupying mass lesion, hemorrhage, hydrocephalus, abscess, or cerebral edema [55]. Children with recurrent complex FSs and other neurological symptoms, such as an abnormal head circumference, a significant developmental delay, or a persistent focal neurological abnormality, should be evaluated using non-urgent MRI.

Blood Culture Analysis

Further testing, including a full blood count, C-reactive protein, urea, calcium, magnesium, glucose, and electrolyte levels, and blood cultures if bacterial sepsis is suspected, should be considered in children aged under one year who present with the first episode of complex FS or have symptoms suggestive of intracranial infection [20]. Other testing options include urine dipstick and culture tests, chest X-rays, stool culture tests, and other diagnostic testing. The multiplex polymerase chain reaction analysis has been important in discovering numerous viruses responsible for FSs in children and how it might help with the risk classification of these patients in the future to reduce unnecessary antibiotic use. Hypoglycemia is a rare finding in children presenting with FSs in the hospital or emergency department; hence, testing the blood glucose levels in this population has very minimal clinical significance. They recommended that such tests be carried out instead on a patient who is actively convulsing after receiving their first dosage of benzodiazepines or on a child whose mental state has been consistently changed [55].

Management 

When a kid has FS, parents may experience severe anxiety, with several believing that the death of their child is going to occur. Stress in the family, such as the youngster sleeping in the parent's bed, might result from worries about the child's future [24]. The anxiety can be reduced, and a return to normal life for the family is possible with reassurance that the child won't pass away and education regarding the cause and outlook for this disorder. Since the seizure has usually ended by the time the kid is examined by a doctor, intervention to stop it is typically not necessary. On the other hand, if the seizure is still going on when the child gets to the hospital, treatment should start right away. If a child has a simple FS and is in good clinical condition and the infection source is obvious, hospitalization is not necessary. Following a period of observation in the emergency department, preferably six hours after the occurrence, the child can be discharged. Most FS episodes are brief and self-terminating, necessitating no long-term antiepileptic medication therapy. Antiepileptic medications should be administered to a kid who is still convulsing when brought to the emergency department if the seizure lasts more than five minutes, is febrile, or is recurrent [28]. Recognizing red flags is crucial when assessing a child with FS because they help determine whether additional care is necessary. The red flag signs of FSs include if a child presents with complex FSs, meningeal signs like Kernig's and Brudzinski's signs are positive, neck stiffness is present, and anterior fontanelle is bulging. In the acute phase, treatment focuses on determining the underlying cause of the fever and treating its symptoms. Encourage the child to drink to ensure that they are getting enough fluids, and you can give them paracetamol or ibuprofen to ease their discomfort from the infection. Prolonged seizures call for immediate treatment. An ambulance should be contacted when a seizure lasts more than five minutes. For a seizure that lasts longer than five minutes, acute therapies such as rectal diazepam (0.5 mg/kg) and buccal (0.4-0.5 mg/kg) or intranasal (0.2 mg/kg) midazolam are effective and can be given at home [20]. When administered orally or rectally during the onset of a febrile illness, diazepam has shown a statistically significant, but clinically modest, capacity to reduce the probability of the occurrence of a FS [14]. An ambulance should be called if the seizure does not end after another 10 minutes, if the child is still twitching after the bigger jerking has ended, or if another seizure starts before the child regains normal consciousness. Midazolam has been found through randomized controlled trials to be more effective than diazepam. Antibiotics should be given for microbiological, febrile infections like tonsillitis, otitis media, or pneumonia [55]. There is no clear evidence that treating simple FSs can stop the later onset of epilepsy, hence it is not advised. Complex FSs are unlikely to stop spontaneously and hence treatment is required [1]. In numerous well-controlled trials, it has been demonstrated that barbiturates, such as phenobarbital, help lower the incidence of recurrent FSs when administered daily at dosages that produce a serum concentration of 15 g/mL or above. Chronic phenobarbital treatment is infrequently recommended since, in most situations, the hazards appear to outweigh the benefits [14]. Both human and animal studies have shown that valproic acid therapy given daily helps lower the likelihood of recurrent FSs. However, it is very infrequently utilized because young children and/or those with neurological abnormalities, who are frequently evaluated for prophylaxis, also have the highest risk of fatal idiosyncratic hepatotoxicity [14]. Other prophylactic antiepileptic drugs, such as phenytoin and carbamazepine, can also be used. There is no evidence available on the effectiveness of the newer antiepileptic drugs, such as gabapentin, lamotrigine, topiramate, tiagabine, or vigabatrin, in the management of FSs [7].

## Conclusions

The most typical variety of seizures in children is FS. Few children experience long-term health issues, and the majority have a favorable prognosis. To properly diagnose FS clinically, intracranial infections must be eliminated, especially in cases of complicated FS. Controlling symptoms and addressing the underlying cause of the fever constitute management. After an FS, parents and caretakers are frequently disturbed and afraid, so it is important to properly educate them on the generally good prognosis and provide them with guidance on how to handle the fever of their child and the acute period of FS. The level of assessment required in children with FSs has significantly changed since the introduction of the *Hemophilus influenza* type B and *Streptococcus pneumoniae* vaccinations. It is essential to categorize FSs according to their duration and other features to determine how to manage and predict them. To avoid the improper use of diagnostic procedures and treatments, pediatricians and neurologists must be well informed on FS care. 
